# Sevoflurane-associated diffuse alveolar hemorrhage: A rare cause of postoperative dyspnea. Case report and literature review

**DOI:** 10.1016/j.rmcr.2026.102513

**Published:** 2026-10-01

**Authors:** Amir Ashiq, Rabia Ahmed, Muhammad Waqas, Fatima Anwar

**Affiliations:** a Hospitalist, Hillcrest Health System, Tulsa, OK, USA; b Department of Medicine Dow International Medical College, DUHS, Karachi, Pakistan; c Hospitalist, Rochester General Hospital, Rochester, NY, USA; d Department of Medicine, Allama Iqbal Medical College, Lahore, Pakistan

## Abstract

Diffuse alveolar hemorrhage (DAH) is a rare, life threatening, early post-operative pulmonary complication of inhaled anesthetics, which are used for induction and maintenance of general anesthesia. It refers to hemorrhage originating from pulmonary microcirculation. It presents with hemoptysis, anemia, diffuse lung infiltration, and acute respiratory failure. Common causes are systemic vasculitis, Collagen vascular disease, Bone marrow transplantation, Drugs, and idiopathic conditions. Treatment ranges from supportive care and withdrawal of offending drugs to high-dose steroids, immunosuppressants, and plasmapheresis. Sevoflurane induced diffuse alveolar hemorrhage is a rarely reported entity in the literature that clinicians should be aware of and consider among the post-operative causes of dyspnea while ruling out other etiologies. We present a literature review over sevoflurane induced diffuse alveolar hemorrhage and a case involving a young otherwise healthy patient who underwent surgery for metatarsal fractures under general anesthesia, but diffuse alveolar hemorrhage developed after sevoflurane inhalation in the postoperative period.

## 1 Introduction

Pulmonary complications are common in the postoperative setting due to a wide array of etiologies. Incidence varies from 5 to 83 percent, depending on various factors [1,2]. Diffuse alveolar hemorrhage is a rare postoperative pulmonary complication that can present with hemoptysis, anemia, diffuse pulmonary infiltrates on imaging, and/or hypoxemic respiratory failure. Among other etiologies, multiple medications have been implicated in causing DAH.

We present a young patient who underwent surgery for metatarsal fractures under general anesthesia, but diffuse alveolar hemorrhage developed after sevoflurane inhalation in the postoperative period.

## 2 Case Presentation

29-year-old male with h/o asthma underwent repair of right 4th and 5th metacarpal fractures under general anesthesia. Postoperatively, while in the recovery area, he started having hemoptysis with a drop in saturation to 87% on RA. He was placed on 3 L of oxygen via nasal cannula. Patient was recommended for hospital admission, but he left against medical advice. He presented to the emergency department a few hours later with worsening shortness of breath and continued hemoptysis. His vitals on arrival included blood pressure of 113/88 mmHg, respiratory rate 24, pulse 129 beats per min, temperature 97 °F, and oxygen saturation of 87% while breathing ambient air. He was placed on 2 L of oxygen by nasal cannula with improvement in saturations to 98%.

Laboratory workup showed a white blood cell count of 20.5, hemoglobin 11.5, platelets 392. No other significant lab abnormalities were noted, including a normal aPTT and INR. Chest X-ray showed widespread bilateral opacities [Fig. 1]. CT pulmonary angiogram did not show a pulmonary embolism. It showed bilateral perihilar dense alveolar opacities concerning for diffuse pulmonary hemorrhage [Fig. 2]. In addition to breathing treatments, the patient was given 2 L of normal saline, vancomycin, and cefepime.

**Fig. 1:**
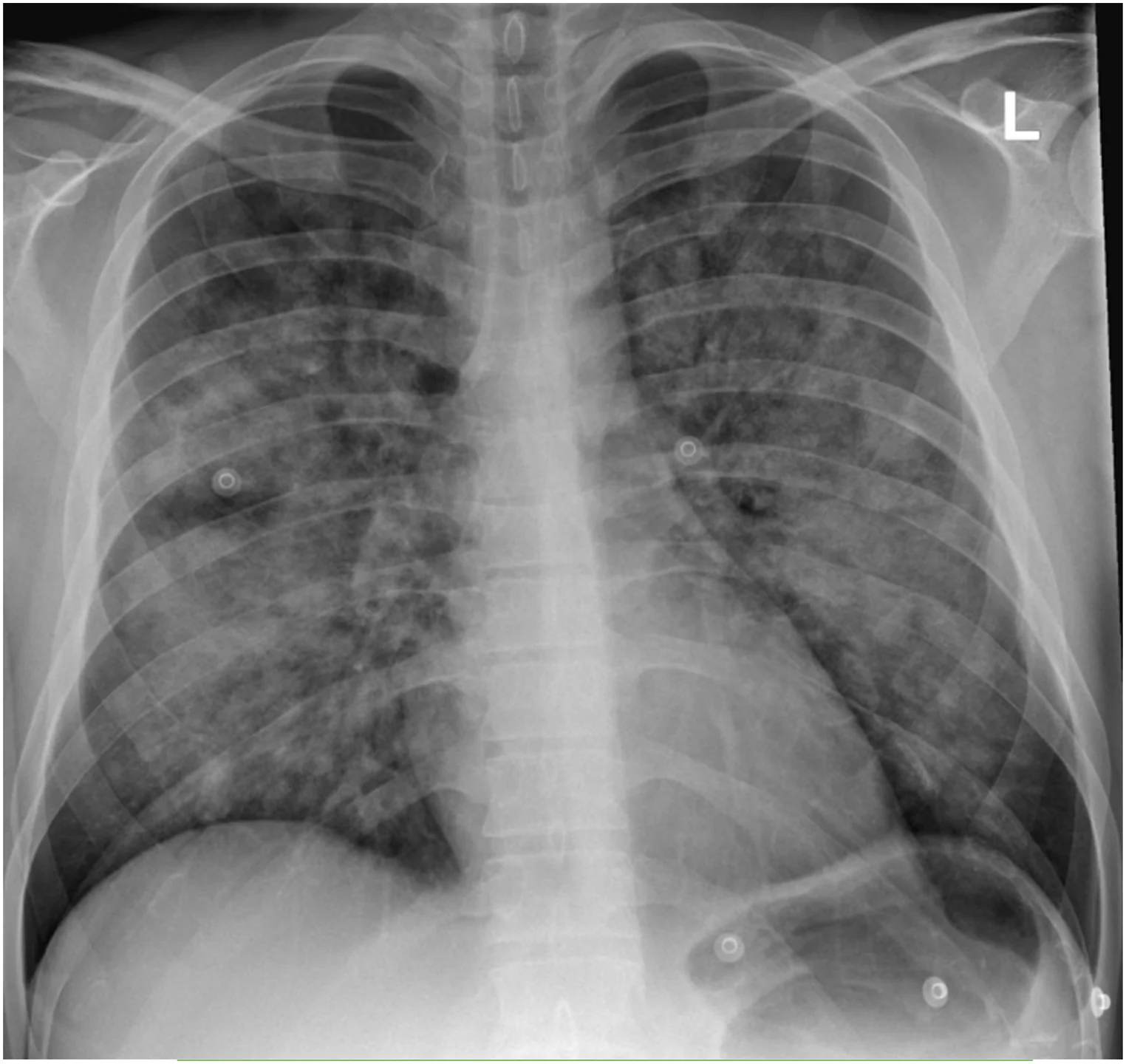
Chest X-ray showing widespread bilateral alveolar opacities.

**Fig. 2:**
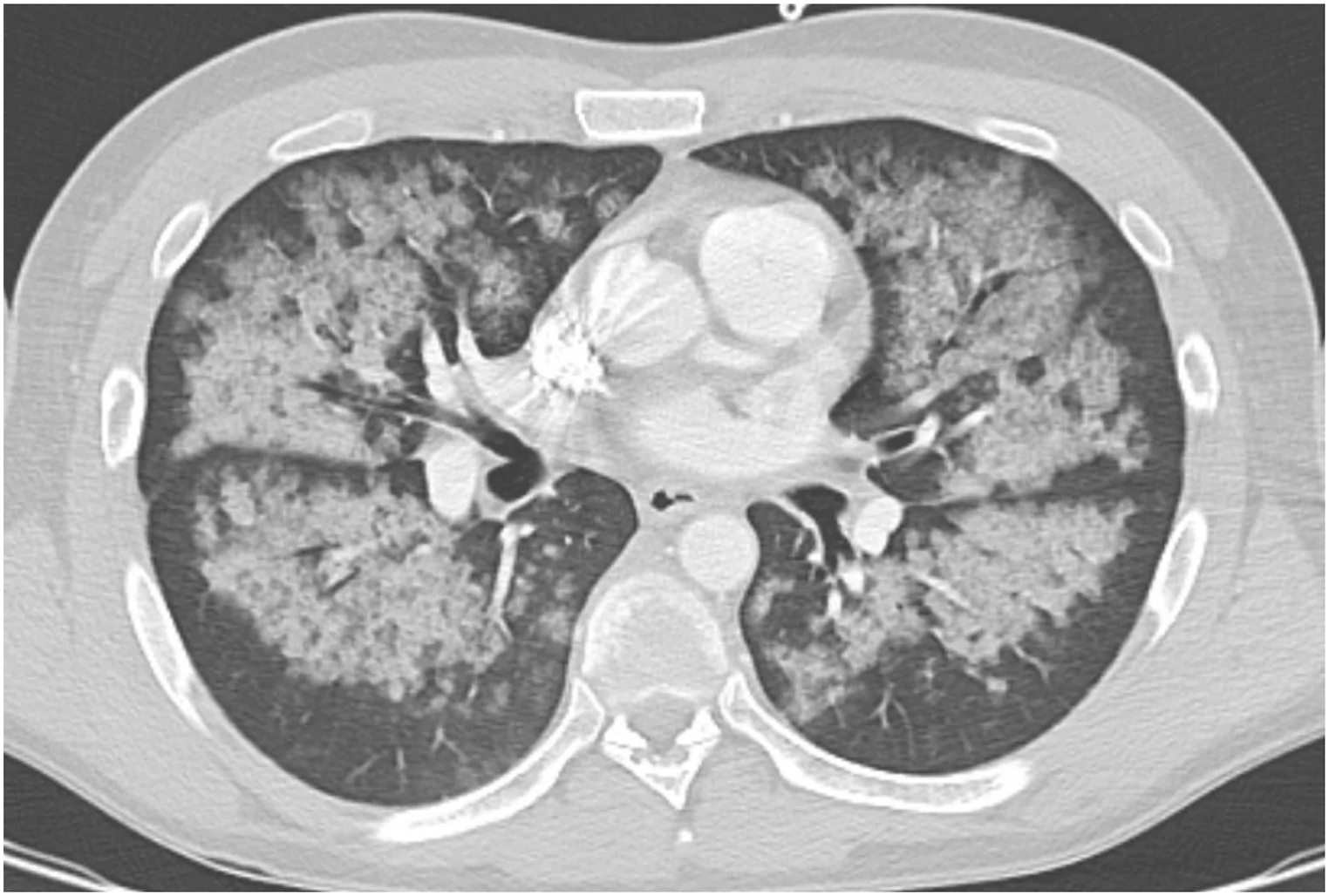
Axiel CT images showing bilateral diffuse central predominant alveolar opacities.

The following day, he was seen by pulmonology. His hemoglobin dropped to 9.1 compared to 11.4 on admission, but clinically the patient had improved. Hemoptysis and shortness of breath had resolved, and he was breathing comfortably with saturations approximating 100% on ambient air. Repeat CXR showed slightly improved bilateral opacities. He admitted to smoking cigarettes and marijuana but denied use of IV drugs. His urine drug screen was consistent with that. He had no history of autoimmune disorders. ANA and ANCA came back negative. The etiology of diffuse pulmonary hemorrhage was unclear on presentation. Review of records revealed that he had received sevoflurane during hand surgery as part of general anesthesia. This was attributed to causing the patient's symptoms. He was offered bronchoscopy, but considering he was doing much better, he did not want to undergo bronchoscopy. Pt continued to do better and was discharged home the following day. Four weeks later, the patient had a CT abdomen/pelvis for nausea, vomiting, and abdominal pain, and imaging of the part of the lungs showed resolution of previously seen infiltrates [Fig. 3].

**Fig. 3:**
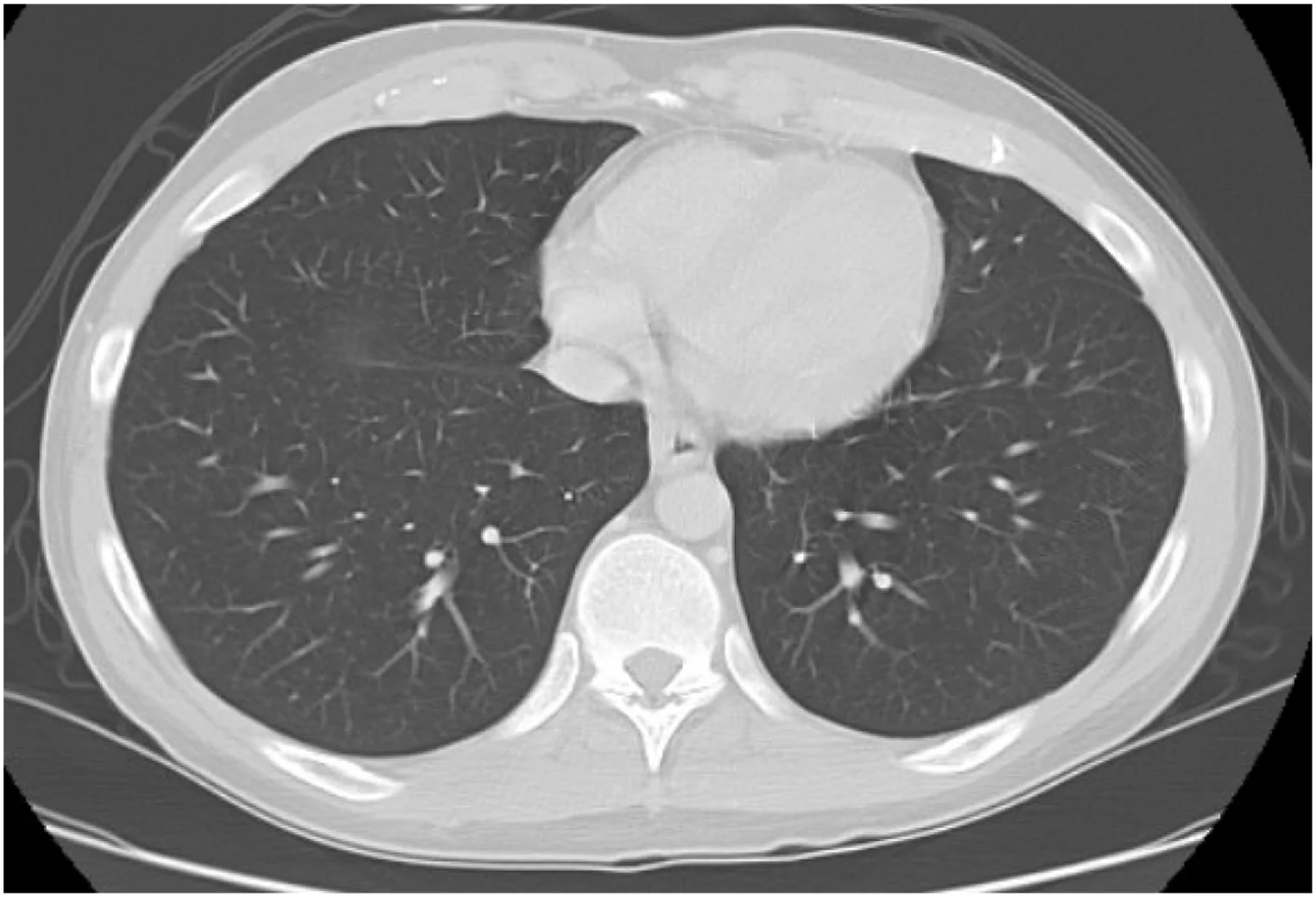
Axial CT images showing resolution of previously seen bilateral lung opacities.

## 3 Discussion

Postoperative airway and pulmonary complications can be divided into immediate (e.g., laryngospasm, upper airway edema), early (occurring in hours to days, e.g., pneumothorax, atelectasis, pneumonia), and late (occurring in weeks to months, e.g., tracheal stenosis).

Diffuse alveolar hemorrhage (DAH) is a rare, early postoperative pulmonary complication of inhalational anesthetics, usually presenting within hours of anesthesia. It's a clinicopathologic syndrome of pulmonary hemorrhage that originates from pulmonary microcirculation. It presents with hemoptysis, anemia, diffuse lung infiltration, and acute respiratory failure. Common causes are systemic vasculitis such as granulomatosis with polyangiitis (GPA), eosinophilic granulomatosis with polyangiitis (EGPA), microscopic polyangiitis, Goodpasture's syndrome, Collagen vascular disease, Bone marrow transplantation, Drugs, and idiopathic conditions.

DAH is associated with pulmonary capillaritis, bland pulmonary hemorrhage, diffuse alveolar damage, and miscellaneous histology. Pulmonary capillaritis is the most common finding [3]. The diagnosis is confirmed by the observation of the accumulation of red blood cells (RBCs), fibrin, or hemosiderin-laden macrophages in the alveolar space on pathologic biopsy. Hemosiderin, a product of hemoglobin degradation, appears at least 48–72 hours after bleeding and is helpful in distinguishing DAH from surgical trauma [4,5]. The treatment of DAH ranges from supportive care and withdrawal of offending drugs to high-dose steroids, immunosuppressants, and plasmapheresis [6].

Sevoflurane is a commonly used inhalational anesthetic for the induction and maintenance of general anesthesia. Its frequently reported respiratory side effects include cough (5–11%), apnea (2–6%), laryngospasm (2–8%), and respiratory depression. Rare but more serious pulmonary complications, including airway thermal injury and acute lung injury/acute respiratory distress syndrome, have also been described [7,8]. Diffuse alveolar hemorrhage (DAH) associated with sevoflurane exposure is particularly uncommon, with only a limited number of cases reported in the literature [[9], [10], [11], [12], [13], [14], [15], [16], [17], [18]]. Table 1 summarizes the reported cases of DAH from inhaled anesthetics. Interestingly all patients were young to middle aged males with median age of 30 years. Severity of DAH was variable among patients with most needing only supplemental oxygen, while some needing reintubation and mechanical ventilation. Glucocorticoids were used in 4 cases and antibiotics were used in only 1 case. All patients had complete recovery followed by hospital discharge. Pathophysiology is unknown, although it is hypothesized that a metabolite of sevoflurane may cause pulmonary endothelial toxicity [12].

**Table 1:** Summarizing reported cases of inhaled anesthetic induced diffuse alveolar hemorrhage.

Author	Age and Gender	Treatment	Outcome
Ashish K Khanna, Kenneth C Cummings	48 M	Reintubation and mechanical ventilation, management like ARDS including high PEEP and low tidal volume	Complete recovery in one week
Kim CA, Liu R, Hsia DW	31 M	Supplemental oxygen, IV methylprednisolone	Complete recovery
Mersh R, Ross C	31 M	Supportive treatment with high flow oxygen	Complete recovery and discharge within 48 hours with normal CXR at 6 weeks follow up
Cengiz O, Kivrak A, Yegen M, Demir M	38 M	Reintubation and mechanical ventilation and IV methylprednisolone 500mg on Day1, 80mg on day 2 and 3	Extubating on Day 3 followed by complete recovery. Resolution of lung infiltrates at 1 month follow up imaging. No recurrence noted at 6 month follow up.
Austin A, Modi A, Judson MA, Chopra A	20 M	High flow oxygen followed by reintubation and mechanical ventilation	Extubation on Day 3. Complete resolution on lung infiltrates at 1 month follow.
Ahmed-Khan M, Moin K, Funk C, Sachdev M, Jiffry MZM	21 M	Supplemental O2, IV methylprednisolone 250mg × 3 days followed by 5 days of prednisone, Vancomycin and Rocephin	Weaned off oxygen with complete recovery
Khan NA, C Mira-Avendano I, Dinh K	20 M	Supportive treatment with supplemental O2	Complete recovery and discharge within 24 hours
Baran Ketencioğlu B, Hastekkeşin M, YetkiN NA, Tutar N	23 M	Supportive treatment with supplemental oxygen, IV methylprednisolone 100mg on Day 1 followed by taper and discontinuation on day 3.	Complete recovery and discharge on day 4, Complete resolution of lung infiltrates on follow up imaging at 1 month, no recurrence at 2 year follow up.
Jordan JH, Pedroza S, Billefals ES, Cruz G	42 M	Supportive treatment with supplemental oxygen	Complete recovery and discharge
İlgün JD, Keskin AT, Ateş S, Güler S	29 M	High flow nasal canula, 1 mg/kg methylprednisolone x 3 days, Single dose of 1g inhaled tranexamic acid	Complete recovery

## 4 Conclusion

Postoperative dyspnea is a frequently encountered complaint. While common etiologies account for most cases, rare causes like DAH should be suspected in any patient with diffuse alveolar infiltrates on chest X-ray, hypoxemia, anemia, and hemoptysis. Careful attention to the medical history, physical examination, and a targeted laboratory evaluation often reveals the underlying cause. Sevoflurane-induced diffuse alveolar hemorrhage is a rare complication of inhaled anesthetics. It typically occurs in younger patients after surgery and is usually managed conservatively. Prompt recognition and management, after ruling out other causes, are essential for optimal patient outcomes.

## CRediT authorship contribution statement

**Amir Ashiq:** Writing – original draft, Validation. **Rabia Ahmed:** Writing – review & editing, Software, Data curation. **Muhammad Waqas:** Supervision. **Fatima Anwar:** Data curation.

## Declaration of Competing Interest

The authors declare that they have no known competing financial interests or personal relationships that could have appeared to influence the work reported in this paper.
